# Self‐Powered Biomimetic Pressure Sensor Based on Mn–Ag Electrochemical Reaction for Monitoring Rehabilitation Training of Athletes

**DOI:** 10.1002/advs.202401515

**Published:** 2024-04-23

**Authors:** Ziyan Yang, Qingzhou Wang, Huixin Yu, Qing Xu, Yuanyue Li, Minghui Cao, Rajendra Dhakal, Yang Li, Zhao Yao

**Affiliations:** ^1^ College of Electronics and Information Qingdao University Qingdao 266071 China; ^2^ Department of Computer Science and Engineering Sejong University Seoul 05006 South Korea; ^3^ School of Integrated Circuits Shandong University Jinan 250101 China

**Keywords:** biomimetic pressure sensor, human health monitoring, hydrogel electrolyte, potentiometry, wearable device

## Abstract

Self‐powered pressure detection using smart wearable devices is the subject of intense research attention, which is intended to address the critical need for prolonged and uninterrupted operations. Current piezoelectric and triboelectric sensors well respond to dynamic stimuli while overlooking static stimuli. This study proposes a dual‐response potentiometric pressure sensor that responds to both dynamic and static stimuli. The proposed sensor utilizes interdigital electrodes with MnO_2_/carbon/polyvinyl alcohol (PVA) as the cathode and conductive silver paste as the anode. The electrolyte layer incorporates a mixed hydrogel of PVA and phosphoric acid. The optimized interdigital electrodes and sandpaper‐like microstructured surface of the hydrogel electrolyte contribute to enhanced performance by facilitating an increased contact area between the electrolyte and electrodes. The sensor features an open‐circuit voltage of 0.927 V, a short‐circuit current of 6 µA, a higher sensitivity of 14 mV/kPa, and outstanding cycling performance (>5000 cycles). It can accurately recognize letter writing and enable capacitor charging and LED lighting. Additionally, a data acquisition and display system employing the proposed sensor, which facilitates the monitoring of athletes’ rehabilitation training, and machine learning algorithms that effectively guide rehabilitation actions are presented. This study offers novel solutions for the future development of smart wearable devices.

## Graphical abstract

A self‐powered pressure sensor utilizing a potential conversion mechanism based on electrochemical reactions is proposed. The proposed sensor incorporates interdigital electrodes and a microstructured electrolyte to increase the sensitive area. Additionally, a data acquisition and machine learning algorithm system are developed using the sensor microcontroller for real‐time monitoring of sports rehabilitation.

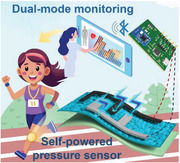

## 1 Introduction

In recent years, rapid progress has been achieved in wearable electronic devices, which have gained significance in smart homes, e‐skin, and health monitoring.^[^ ^1^, ^2^, ^3^, ^4^, ^5^, ^6^ ^]^ An urgent need exists to implement quantifiable and scientifically guided monitoring systems to capture data on athletes’ static and dynamic motion modes. To address this issue, researchers have developed a diverse range of dual‐mode pressure sensors. Numerous capacitive^[^ ^7^, ^8^, ^9^, ^10^, ^11^ ^]^ and resistive^[^ ^12^, ^13^, ^14^ ^]^ sensors have been proposed to detect static and dynamic stimuli; however, they rely on batteries or external power sources, which are not flexible or convenient for deployment in many sports scenarios. Self‐powered sensors have significant advantages because they do not require frequent battery replacements or maintenance.^[^ ^15^, ^16^, ^17^, ^18^ ^]^ To achieve self‐powered dual‐mode sensing, researchers have integrated piezoelectric/triboelectric sensors with capacitance/resistance sensors.^[^ ^19^, ^20^, ^21^, ^22^ ^]^ However, the integrated sensors are generally complex to manufacture, and the interaction between different types of sensors may lead to interference in signal transmission, thus affecting the accuracy of the measured data. Researchers have also explored a new self‐powered sensing mechanism, the potentiometric conduction mechanism,^[^ ^23^ ^]^ which draws inspiration from skin cells’ ability to sense external stimuli and generate membrane potentials. Specifically, pressure signals are encoded into sustainable electrical signal outputs using redox reactions between the two electrodes of the pressure sensor,^[^ ^24^, ^25^, ^26^, ^27^ ^]^ enabling both self‐powering capability and integrated detection of static and dynamic stimuli.

The sensing mechanism of a potentiometric pressure sensor closely resembles that of a primary battery. When electrodes of two different materials come into contact with a solid electrolyte, redox reactions occur owing to the different electron affinities of the electrodes, resulting in a potential difference. By manipulating the contact between the electrodes and electrolyte, an external mechanical force can be transformed into a potential difference as the output. Kim et al. reported an electrochemical‐based self‐powered pressure sensor,^[^ ^28^ ^]^ comprising two electrodes and a functional sponge with a liquid electrolyte. However, the liquid electrolyte has the disadvantage of an unstable output. Zhang et al. reported a zinc‐ion battery‐type pressure sensor^[^ ^29^ ^]^ with a sandwich electrode structure in which the anode was separated from the electrolyte using a nanofiber barrier. Lei et al. demonstrated a sandwich‐structured pressure sensor^[^ ^30^, ^31^ ^]^ based on hydrated graphene oxide as the solid electrolyte and a nanofiber insulation layer to achieve a broad pressure‐sensing range. One disadvantage of using a sandwich structure is that it requires an additional isolation layer and involves complex processing steps. Wu et al. reported two hydrogel‐based potentiometric sensors^[^ ^32^, ^33^ ^]^ with side‐by‐side electrode structures and lattice‐like microstructures fabricated on a hydrogel electrolyte. One of the sensors achieved potentiometric‐triboelectric hybridization, whereas the other responded to pressure and temperature. However, this device produced a low output voltage. The voltage range between the oxidation and reduction reactions, known as the electrochemical window,^[^ ^34^ ^]^ can determine the thermodynamic stability of an electrolyte. Solid‐state electrolytes have a superior electrochemical window compared to liquid electrolytes. For this reason, we chose a solid‐state hydrogel electrolyte to improve the stability of the sensor. Additionally, the Faraday current of an electrochemical reaction is proportional to the electrode area, the concentration of redox‐active substances, and the number of electrons transferred.^[^ ^35^, ^36^ ^]^ Therefore, increasing the contact area between the electrolyte and electrodes accelerates the kinetics of electrolyte decomposition. It could lead to effective electron collection from the oxidation of the electrolyte, hence enhancing the oxidation current.

In this study, we utilized a laser‐induced graphene (LIG) substrate for screen‐printing the electrodes (anode and cathode). A microstructured hydrogel‐based solid‐state electrolyte to realize a potentiometric sensing mechanism was developed, leveraging an external stimulus to modulate the output voltage for monitoring motor rehabilitation. In particular, we designed a primary battery system comprising interdigital electrodes composed of MnO_2_ and Ag as the cathode and anode, respectively, and a hydrogel electrolyte based on polyvinyl alcohol (PVA) and phosphoric acid (H_3_PO_4_). The working mechanism, performance, and potential applications of the resulting sensor were investigated systematically. Using the PVA‐based solid‐state electrolyte combined with MnO_2_ and Ag electrodes enhanced the output voltage and eliminated the need for an additional isolation layer because the sandpaper‐like microstructure of the electrolyte generated an air isolation layer. The developed potentiometric sensor offers several advantages, including fast response, durability, and the ability to monitor both static and dynamic stimuli. Additionally, the pressure sensor does not need external power modules. This facilitates device miniaturization and integration, particularly in space‐constrained applications. Furthermore, the sensitivity and maximum output voltage of the sensor could be modified by adjusting the microstructure and water content of the PVA hydrogel. In addition to monitoring muscle activity and movement in the human body, the electrochemical pressure sensor can also charge capacitors, illuminate light‐emitting diodes (LEDs) and electronic watches. Using this sensor, a rehabilitation‐monitoring system for athletes and individuals with suboptimal health was developed. This system includes a data acquisition and display system and a machine‐learning algorithm system. The machine‐learning algorithm system effectively monitors and accurately identifies various rehabilitation actions using the collected data. This study offers novel solutions for the future development of smart wearable devices and self‐powered mechanical sensors.

## 2 Results and Discussion

### 2.1 Sensor Preparation, Sensing Mechanism, and Rehabilitation System

**Figure 1:**
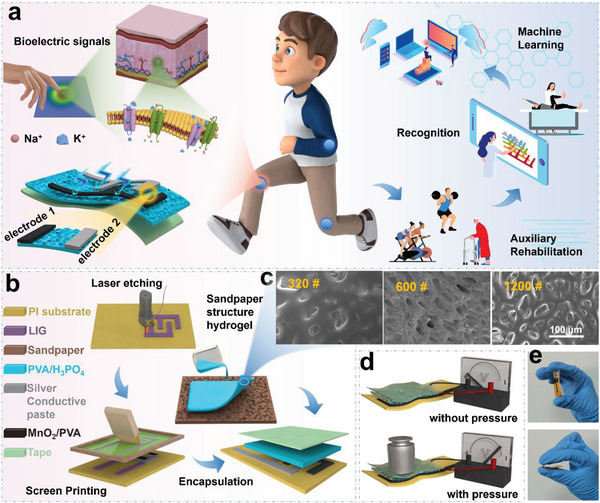
Preparation of a potentiometric sensor using an electrochemical reaction mechanism to construct a monitoring system for exercise rehabilitation. a) Potentiometric sensors inspired by skin attached to body parts to monitor exercise rehabilitation. b) Fabrication process of the sensor. c) SEM images of the surfaces of hydrogels produced using 320‐, 600‐, and 1200‐mesh sandpaper samples as substrates. d) Illustration of an output voltage produced by the sensor when an external force is applied. e) Photographs and bending of the potentiometric sensor.

**Figure** **1**a illustrates how the skin senses external stimuli and generates a membrane potential through sodium‐potassium channels. Inspired by this bioelectrical signal transmission, we developed a self‐powered, potentiometric, biomimetic pressure sensor for monitoring sports rehabilitation. Figure 1b outlines the sensor fabrication process. First, the interdigital electrodes were realized using LIG on a polyimide (PI) film.^[^ ^37^, ^38^ ^]^ Subsequently, the MnO_2_ precursor and conductive silver paste were coated on the interdigitate graphene electrode using a screen‐printing technique to serve as cathode and anode, respectively. For electrolyte preparation, a mixture of H_3_PO_4_ and PVA was poured onto sandpaper to form a hydrogel with a specific microstructure. The so‐formed hydrogel, with a thickness of ≈61 µm, was used as the solid electrolyte (Figure S1, Supporting Information). The electrodes and hydrogel electrolytes were then encapsulated with adhesive tape to create a potentiometric sensor. Scanning electron microscopy (SEM) images of the hydrogels produced with sandpaper of different mesh sizes are shown in Figure 1c.

When a mechanical force is applied to the sensor, an electrochemical reaction occurs between the hydrogel electrolyte and electrode, resulting in a potential difference between the two electrodes (Figure 1d). The potentiometric sensor produces a voltage signal due to redox reactions involving manganese and silver ions in the respective electrodes, and the discharge process can be represented as

(1) $$\mathrm{Electrolyte}:H_{3}PO_{4}\to H_{2}PO^{4-}+H^{+}$$

(2) $$\mathrm{Anode}\left(+\right):\mathrm{Mn}O_{2}+H^{+}+e^{-}\to \mathrm{MnO}\left(\mathrm{OH}\right)$$

(3) $$\mathrm{Cathode}\left(-\right):\mathrm{Ag}+Cl^{-}\to \mathrm{AgCl}\;+\;e^{-}$$

Because of the sandpaper‐like surface microstructure of the hydrogel electrolyte, in the absence of an external force, this design significantly limits the contact area between them, resulting in a zero output voltage. When an external force is applied, the contact area between the electrolyte and electrode increases, resulting in a larger output voltage. The maximum output voltage and sensitivity of the fabricated flexible sensor could be modulated by adjusting the water content in the electrolyte and the mesh size of the sandpaper used to prepare the hydrogel electrolyte. In addition, the sensor could be bent without compromising its performance after recovery (Figure 1e).

### 2.2 Simulation and Characterization of the Sensor

**Figure 2:**
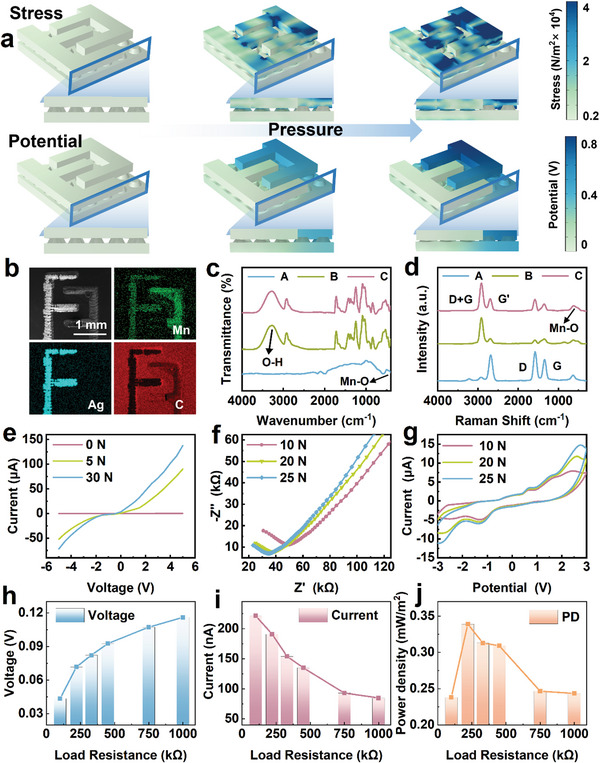
Simulation and characterization of the potentiometric sensor. a) Finite element simulation of the mechanical and electrochemical properties of the potentiometric sensor. b) SEM and EDS images of the interdigital electrodes. c,d) FTIR and Raman spectra of three electrode samples: the MnO_2_/C/DMF electrode (Sample A), MnO_2_/C/PVA electrode (Sample B), and MnO_2_/C/PVA electrode after discharge (Sample C). e) *I–V* curves of the sensor. f) EIS and g) *C–V* curves of the sensor. h–i) Output voltage and output current under different loads. j) Power density under varying loads.

Finite element simulations were conducted to analyze the mechanical and electrochemical properties of the sensor. The deformation of the hydrogel microstructure and the variation in the contact area between the electrolyte and electrodes with an increase in the applied mechanical force were investigated. A progressive increase in the potential difference between the two electrodes was observed with increasing pressure (**Figure** **2**a). The simulation outcomes agreed well with the experimental observations.

Figure 2b presents the SEM and energy‐dispersive X‐ray spectroscopy (EDS) images of the interdigital electrodes, demonstrating that the electrode ink coverage was adequate. The elemental analyses of Mn, Ag, and C further confirmed the distribution of Mn and Ag on the relevant interdigital electrodes. C was uniformly distributed on the substrate and Mn electrodes.

Two methods were employed to prepare the MnO_2_ ink used to form the cathode of the sensor. In the first method, a MnO_2_ powder was dispersed in dimethylformamide (DMF) through ultrasonication, and the resulting mixture was printed on the interdigital graphene electrode (Sample A). In the second method, the MnO_2_ powder was dispersed in an aqueous PVA solution and subsequently printed on the interdigital graphene electrode (Sample B). Fourier transform infrared (FTIR) and Raman spectroscopic analyses were performed on the cathodes fabricated using the two methods and the discharged MnO_2_/PVA cathode (Sample C). Figure 2c presents the FTIR spectra of the electrodes. Compared to the references,^[^ ^39^, ^40^ ^]^ a band at 3400 cm^−1^ corresponds to O‐H stretching vibrations. Samples B and C exhibit a relatively strong peak at 3400 cm^−1^, consistent with the higher water content of the PVA solution. In accordance with the literature,^[^ ^41^, ^42^ ^]^ in the spectral range of 1000–2000 cm^−1^, vibrational peaks were appeared, representing the interactions of Mn with surrounding materials, such as O‐H, O, and H^+^. The electrochemical activity of a material increases with the intensity of the peak in this range. Thus, Figure 2c reveals that Sample B is more electrochemically active than Sample A. Consequently, Sample B was selected as the cathode in this study. In the (400 to 1000) cm^−1^ range, a characteristic peak that demonstrates a distortion of the Mn octahedron of MnO_2_ is observed.^[^ ^43^ ^]^ This peak weakened and shifted to the low wavenumber range after discharge.

Figure 2d shows the Raman spectra of the cathodes, revealing four characteristic peaks of graphene:^[^ ^44^ ^]^ the D+G peak at 2950 cm^−1^, G′ peak at 2700 cm^−1^, G peak at 1580 cm^−1^, and D peak at 1300 cm^−1^. The cathode comprised LIG coated with MnO_2_ ink. The G and D peaks of Sample A are significantly stronger than those of Sample B, suggesting that the surface coverage of Sample B was superior to that of Sample A. The D+G and G′ peaks can provide information on the stacking structure of graphite.^[^ ^45^ ^]^ Thus, the comparison of the Raman spectra of samples A and B indicates that PVA doping altered the stacking order of graphite. The Raman band of the Mn‐O lattice is observed at 670 cm^−1^.^[^ ^46^ ^]^ After discharge, the peak at 840 cm^−1^ disappeared, and the peak at 630 cm^−1^ intensified.

Next, the electrochemical properties of the sensors were investigated. Figure 2e shows the *I–V* curve of the sensor under different pressures. As the external mechanical force was increased from 0 to 30 N, the total resistance of the sensor decreased, resulting in an increase in the output voltage. Notably, the slope of the curve changed substantially as the load was increased from 0 to 5 N, and a smaller change in slope was observed for the curves recorded at 5 and 30 N. This observation suggests that the total resistance of the device varied more significantly at low pressures compared with that at higher pressures. Figure 2f shows the electrochemical impedance spectra (EIS) of the sensor. The arcs in the mid‐frequency range predominantly reflect the electrochemical processes occurring at the electrolyte interface, whereas the low‐frequency tail primarily indicates ionic diffusion in the active material of the cell electrode.^[^ ^47^ ^]^ The intersection of the tail extension line with the x‐axis reflects the internal resistance, further confirming that the total resistance of the sensor decreased with increasing pressure.

Figure 2g displays the cyclic voltammetry (CV) curves of the sensor. The area of the CV curve increased with increasing pressure, indicating a larger electrochemical surface area of the electrodes at higher pressures. This outcome implies an increased contact area between the electrolyte layer and electrodes with increasing pressure. A constant current charge and discharge test was conducted, and the result is shown in Figure S2 (Supporting Information). Various resistors were employed as external circuit loads to evaluate the output power of the sensor; with an increase in the resistance from 100 kΩ to 1 MΩ, the voltage across the resistor increased, whereas the current decreased (Figure 2h,i). The power density (*P*) of the output is calculated as follows:

(4) $$P\;=\frac{UI}{RS}\;$$

where *U* is the voltage across the resistor, *I* is the resistor current, *R* is the load resistance, and *S* is the effective area of the device (i.e., the area of contact between the electrodes and hydrogel electrolyte layer, which is 40.4 mm^2^). As shown in Figure 2j, the maximum power density of 0.34 mW m^−^^2^ was achieved at a 220 kΩ load.

### 2.3 Mechanical Sensing Properties of the Sensors

**Figure 3:**
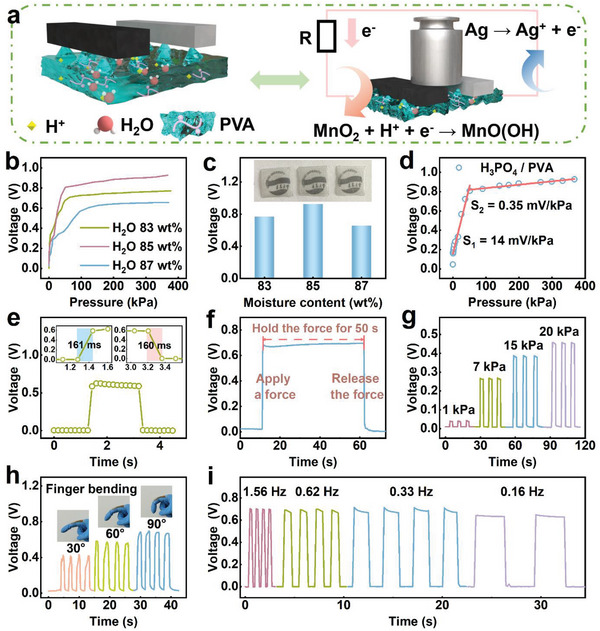
Mechanical sensing performance of the sensor. a) Schematic of the electrochemical mechanism of the sensor. b) Sensitivity (voltage vs pressure) curves, and c) maximum output voltages of the sensors with hydrogel electrolytes with different water content. d) Sensitivity curve of the sensor. e) Response and recovery times of the sensor. f) Response of the sensor to continuous pressure for 50 s. g) Sensor response under different mechanical pressures. h) Sensor response to 30°, 60°, and 90° finger flexion. i) Sensor response to dynamic mechanical stimulation at different frequencies.

The electrochemical mechanism of the sensor was explored (**Figure** **3**a). The PVA hydrogel electrolyte contained H_3_PO_4_, which hydrolyzes to yield H^+^. Because the surface of the hydrogel was modified by sandpaper‐like microstructures, which creates an air isolation layer between the electrode and electrolyte. In the absence of external force, this design significantly limits the contact area between them, resulting in minimal output voltage in a static state. Consequently, this hydrogel solid electrolyte can be isolated from the electrode at zero pressure. Upon applying an external force, the hydrogel contacts the electrode, leading to the reaction of the Mn electrode with H^+^. Specifically, Mn^4+^ gains electrons to form Mn^3+^, whereas Ag loses electrons to form Ag^+^. The electrons then flow in an ordered fashion from the Ag electrode to the Mn electrode, resulting in current flow from the positive electrode to the negative one.

It is possible to tune both the maximum output voltage and the sensitivity of the device by varying the water content of the hydrogel (Figure 3b) and adjusting the density of the hydrogel microstructure (Figure S3a, Supporting Information). The impact of the interdigital electrode size (3, 6 and 9 mm) on sensor's performance was also investigated (Figure S4, Supporting Information). In this case, the sensitivity of the device can be defined as:

(5) S=ΔV/ΔP

Varying the water content of the PVA hydrogel preparation solution (83, 85, and 87 wt.%) resulted in respective device output voltage maxima of 0.67, 0.927, and 0.655 V, respectively (Figure 3c). Further, altering the mesh size of the sandpaper template (320, 600, and 1200 mesh) used for preparing the hydrogel led to output voltage maxima of 0.783, 0.927, and 0.863 V, respectively. The microstructural density of the hydrogel influenced the sensitivity of the device in the first stage of the curve (0 to 35 kPa) (Figure S2b,c, Supporting Information). Hydrogels with a sandpaper‐like microstructure were less transparent than unstructured ones. Based on the output voltage, the hydrogel with a water content of 85 wt.% was prepared using 600 mesh sandpaper as the template, and an interdigital electrode with a 3 mm electrode length was selected for subsequent experiments (Figure 3d). The curves for this condition have good linearity, with a sensitivity of 14 mV/kPa in the first stage (0 to 50 kPa) and 0.35 mV/kPa in the second stage (50 to 380 kPa).

The hysteresis (*ξ*) of a pressure sensor can be calculated by:

(6) $$\xi =\frac{\left|S_{1}-S_{2}\right|}{S_{1}}\times 100\%$$

where *S*_1_ and *S*_2_ are the integral area of the pressure response curve for the loading and unloading process, respectively. According to Figure S5 (Supporting Information) and Equation (6), the hysteresis (*ξ*) of our sensor is calculated as 0.106%, which shows well performance of the hysteresis. The sensor exhibited a good response/recovery performance, with an approximate time of 161/160 ms, respectively (Figure 3e). It could respond consistently to static stimuli. An almost constant voltage was generated when the device was pressed for 50 s (Figure 3f). Moreover, the sensor's performance was thoroughly investigated through continuous pressing test conducted for nearly 10 h (Figure S6, Supporting Information). The sensor maintained a good stable output for ≈8.5 h. Further, the sensor was robust, exhibited high stability in the low‐voltage range (Figure 3g), and responded effectively to dynamic stimuli at various low frequencies (Figure 3i). Moreover, the sensor demonstrated remarkable durability, cycling under a mechanical force of 15 kPa for 5000 cycles (over 10 000 s) without any discernible shift in output (Figure S7, Supporting Information). The sensor exhibits good long‐term stability, and the voltage drops within 0.1 V after seven days by continues testing (Figure S8, Supporting Information).

In addition, the potentiometric sensor is versatile and can detect a broad spectrum of human activities. When attached to finger joints, it could discern various degrees of finger flexion (Figure 3h). Moreover, it could effectively detect the flexion of the wrist, elbow, and knee (Figure S9a–c, Supporting Information). The sensor itself could be bent and released, resulting in a prompt response and recovery (Figure S9d, Supporting Information). It responded to air blowing, indicating its sensitivity to even small pressure variations (Figure S10a, Supporting Information). Placing the sensor on the sole of the shoe enabled differentiation between walking and running motions (Figure S10b, Supporting Information). Impressively, the sensor could be utilized for Morse code input (Figure S10c, Supporting Information), and tapping the letters “QDU” on the sensor produced a well‐represented waveform. These results indicate the capability of our sensor to respond to both dynamic and static stimuli.

Separate tests were conducted for humidity and temperature (Figure S11, Supporting Information). When humidity ranged from 30% to 70%RH, the output voltage varied weakly, within 0.01 V. The sensor package may be more properly insulated so that the effect of humidity on the sensor is negligible. However, when the temperature was varied from 20 to 100 °C, the voltage changed by ≈0.03 V per 10 °C. It is possible that the increase of temperature accelerated the chemical reaction inside the sensor, increasing the output voltage. The sensing performance (voltage, current, power density, and sensitivity under low voltage) of the sensor in this study was compared to that of the currently reported state‐of‐the‐art potentiometric pressure sensors (Table S1, Supporting Information). It can be seen that the proposed sensor show higher output voltage and better sensitivity.

### 2.4 Sensors for Identification Writing and Self‐Powered Applications

**Figure 4:**
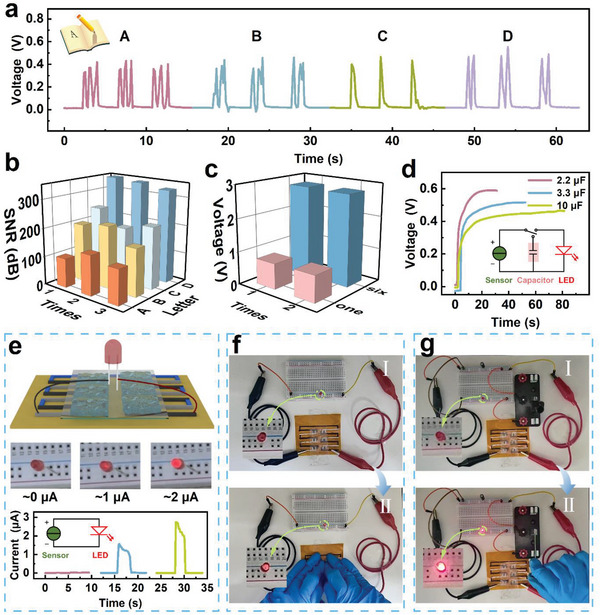
Potential applications of the proposed sensor in recognizing letters, lighting LEDs, and charging. a) Sensor response to writing different letters. b) Signal‐to‐noise ratio for different letter waveforms. c) Voltage output response of a single unit and six units in series. d) Charging curves of the sensor for capacitors of different capacitances. e) Schematic of the device array and the brightness of the LED at different pressures and the corresponding currents. f) Illustration of using a sensor array to turn on an LED by pressing it, and g) turning an LED on by charging the capacitor.

The sensor could precisely recognize written alphabets (A, B, C, and D), as illustrated in **Figure** **4**a. Each letter elicited a distinctive waveform response, which could be deduced from the number of waveforms that corresponded to the number of strokes in the letter. Different letters could be clearly distinguished. Figure 4b illustrates the repeated writing of the letters “ABCD” three times and presents the signal‐to‐noise ratio (SNR) of the output waveform, calculated using the following formula:

(7) $$SNR\;=\frac{P_{Signal}}{P_{Noise}}\;={\left(\frac{A_{Signal}}{A_{Noise}}\right)}^{2}\;$$

(8) $$\;SNR_{dB}=10\;log_{10}\left(SNR\right)$$

where *P_Signal_* denotes the power intensity of the signal, *P_Noise_* is the power intensity of the noise, and *A_Signal_* and *A_Noise_* are the peak voltage amplitudes of the signal and noise, respectively.^[^ ^48^ ^]^ We used the SNR ratio to evaluate the device performance,^[^ ^49^ ^]^ as it serves as an indicator for assessing device performance in practical applications. Although the SNR values corresponding to three repetitions of the same letter varied slightly, the average SNR consistently exceeded 100 dB. This result indicates that the output voltage of the sensor significantly surpasses the noise voltage, ensuring high‐quality output waveforms, robust anti‐interference capability, and good overall performance.

The serial connection of six sensors resulted in voltage multiplication (Figure 4c). When a 1 kg weight was placed on the sensor, the output voltage was 0.7 V. However, the output voltage of the six devices in series was not six times the voltage of an individual sensor; instead, it was only 2.8 V. This result may be attributed to the high internal resistance of the devices, which caused losses during the electronic transmission process. The current for a single device was ≈6 µA, but the current for six devices decreased to ≈2 µA (Figure S12, Supporting Information). In addition, the sensor units could be employed to charge capacitors. A single sensor could charge capacitors of different capacitances. Specifically, a 2.2 µF capacitor could be charged to saturation in 30 s, and 3.3 and 10 µF capacitors required 50 and 80 s, respectively, to be charged to saturation (Figure 4d). The sensor array could be used to illuminate an LED and control its brightness level. Figure 4e shows the relationship between the brightness of the LED and current. When the sensor array was directly connected to both ends of the LED lamp with wires, the LED lamp could be turned on or off by controlling the pressure applied (Figure 4f). Further, a capacitor powered by the sensor was connected to a single‐knife double‐throw switch and an LED lamp, and the LED lamp was turned on or off by controlling the single‐knife double‐throw switch (Figure 4g; Video S2, Supporting Information). As shown in Figure 4g, step I involves charging the capacitor by pressing the device array, lifting the switch, and keeping the LED off. Step II involves closing the switch, causing the LED to light up. Further, the device array could also be used to display the international common distress signal “SOS,” where the signal light exhibited three short flashes, followed by three long flashes and three short ones (Video S1, Supporting Information). It could also power small electronic meters (Video S3, Supporting Information).

### 2.5 Rehabilitation Monitoring With the Sensor

**Figure 5:**
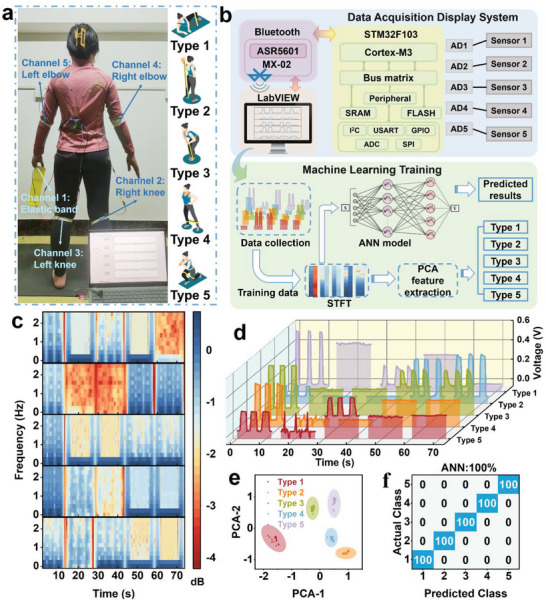
Application of the sensor in rehabilitation monitoring. a) Illustration of the exercise rehabilitation monitoring system attached to the body. b) Schematic of the rehabilitation monitoring system, including a block diagram of the five‐channel data acquisition system and machine‐learning flowchart. c) STFT data processing of waveforms for five actions. d) Waveform diagrams of five different types of five‐channel exercise rehabilitation movements: mussel‐type opening and closing (Type 1), arm flexion and extension (Type 2), bent‐over rowing (Type 3), side leg raising (Type 4), and lunge squat (Type 5). e) Plots of the PCA algorithm. f) Plots of the prediction results of the ANN algorithm.

In sports rehabilitation, the convergence of medicine and athletics seeks to aid individuals with suboptimal health and athletes engaged in prolonged training by restoring muscle and joint functionality, thereby enhancing their athletic performance.^[^ ^50^ ^]^ Five common exercise rehabilitation movements with elastic bands, viz., mussel‐type opening and closing (type 1), arm flexion and extension (type 2), bent‐over rowing (type 3), side leg raising (type 4), and lunge squat (type 5), were selected for assessment. Each movement was tested with five sensors. The five sensors were strategically affixed to the limb joints and areas of the elastic band in contact with the body, thereby establishing five channels that were subsequently connected to a data acquisition module (**Figure** **5**a). The sensors were integrated with a microcontroller, Bluetooth, and LabVIEW programming system to develop a comprehensive data acquisition and algorithmic learning system (Figure 5b). This system enables the monitoring of movement rehabilitation actions, laying the foundation for a medical training integration platform.

In the data acquisition process, the output voltage data generated by the body movements were collected with the ADC module and transmitted to the computer via a Bluetooth chip. The waveforms of different channels were visualized using LabVIEW (Figure S13,  Video S4, Supporting Information). The acquired data were further processed using a short‐time Fourier transform (STFT) to extract the eigenvalues. Principal component analysis (PCA) was then performed for movement classification, and various machine‐learning algorithms, including Artificial Neural Network (ANN), Random Forest, and Support Vector Machines (SVM), were utilized for movement prediction. This multifaceted approach provides essential data for health professionals and coaches to predict the physical injuries resulting from athletic training regimens.

The unique voltage characteristics of each movement, resulting from distinct force points, strengths, and durations, were evident in the data waveforms of the five movements across the five channels (Figure 5d). Applying the STFT allowed the analysis of the time‐ and frequency‐domain characteristics, enabling the extraction of pertinent eigenvalues (Figure 5c). Machine learning algorithms demonstrated robust performance in predicting different rehabilitation maneuvers, with the ANN and Random Forest algorithms achieving 100% accuracy each (see Figure 5f) and the SVM algorithm achieving 92% accuracy (Figure S14, Supporting Information). Additionally, the PCA revealed distinct clustering characteristics for different movements (Figure 5e), emphasizing the high feasibility and efficacy of the intelligent monitoring system in enabling movement rehabilitation, thereby offering valuable guidelines for rehabilitation training.

## 3 Conclusion

A self‐powered potentiometric pressure sensor that utilizes electrochemical reactions was developed to detect both dynamic and static stimuli. The interdigital electrodes of the sensor were prepared using MnO_2_/PVA as the cathode and conductive silver paste as the anode. A hydrogel electrolyte layer featuring a sandpaper‐like structure derived from a sandpaper template was utilized. The effects of the contact area between the hydrogel electrolyte layer and electrodes on the maximum output voltage and sensitivity of the device were investigated. The device exhibited remarkable characteristics, including a high output voltage, high and adjustable sensitivity, a fast response time, and cyclic stability. Its application extends to detect dynamic and static stimuli, such as human physiological signals and muscle movement. A sports rehabilitation monitoring system employing machine‐learning algorithms was developed to classify and identify different rehabilitation postures. This system provides more accurate training guidance data for personalized rehabilitation programs, benefiting athletes and individuals with suboptimal health. Moreover, it lays the groundwork for developing a training–medicine integration platform. The proposed pressure sensor has valuable applications in both healthcare monitoring and sports rehabilitation. It also provides innovative opportunities for the future development of smart wearable devices.

## 4 Experimental Section

### Preparation of Interdigital Electrodes

Interdigital electrodes with a line width of 1.2 mm and spacing of 1 mm were fabricated on a PI film (125 µm) using a laser engraver (KB‐K3020). The MnO_2_ electrode ink was prepared using two methods. In method 1, a MnO_2_ powder (1 g) purchased from Tianjin Dengfeng Chemical Reagent Factory was suspended in DMF (10 g; Shanghai Aladdin Biochemistry Science and Technology Co., Ltd.) and shaken for 15 min in an ultrasonic cleaner (YM‐0105) until the powder was uniformly dispersed in DMF. In method 2, PVA (1 g; Shanghai Aladdin Biochemical Science and Technology Co., Ltd.) was added to deionized water (9 g) and stirred at 100 °C for 2 h on a heated magnetic stirrer (CMVC‐M003H). The resulting mixture was then placed in an electric blast‐drying oven (XCYB‐XGQ2000) and heated at 90 °C for 1 h until the PVA dissolved completely. Then, the MnO_2_ powder (1 g) was added to the PVA solution and stirred at room temperature (25 °C) for 30 min to form a homogeneous suspension. A conductive silver paste was used to form the Ag electrode. A screen‐printed plate with the electrode template was placed on the laser‐etched interdigital electrodes, and one of the electrode pairs was coated with MnO_2_ ink and the other with conductive silver paste. The assembly was then cured for 20 min at 100 °C on a heated bench.

### Preparation of the Hydrogel Electrolyte Layer

PVA (1 g), H_3_PO_4_ (0.8 g; Sinopharm Chemical Reagent Co., Ltd.), and deionized water (9 g (83 wt.%), 10 g (85 wt.%), or 11 g (87 wt.%)) were mixed and stirred using a heated magnetic stirrer at 100 °C for 2 h until the PVA was completely dissolved. The resulting H_3_PO_4_/PVA solution was poured onto a mesh sandpaper with a mesh size of 320, 600, or 1200 and maintained at room temperature (25 °C) for 24 h. Thereafter, the H_3_PO_4_/PVA hydrogel was peeled off using tweezers for use as the electrolyte. The hydrogel electrolyte was cut into squares of a suitable size (typically, 1 cm × 1 cm) for further use.

### Packaging of Devices and Preparation of Device Sets

The prepared H_3_PO_4_/PVA hydrogel was aligned on the surface of the interdigital electrodes and then encapsulated using an adhesive tape (Biaxially Oriented Polypropylene material, Nanjing Baiyi Packaging Material Co., Ltd.). Six linked interdigital electrodes were fabricated via laser engraving. The positive and negative electrodes were screen printed and cured separately. Six pieces of the hydrogel electrolyte were placed on the electrodes and then encapsulated using adhesive tape. A digital multimeter (DMM6500, KEITHLEY) was used to verify the connectivity between the six devices. In the rehabilitation monitoring system, the two electrodes were connected by Dupont wires, fixed with conductive tape (conductive non‐woven fabrics, Shunsheng Electronic Technology Co., Ltd.).

### Machine Learning Training

The collected data were imported into MATLAB software, and three neural network algorithms (ANN, Random Forest, and SVM) were employed for data analysis. The ANN uses 25‐dimensional data as input. After being processed by two hidden layers, five action results were achieved. The study integrated five types of action data, partitioning three‐quarters for training and one‐quarter for testing, followed by normalization of the training data. The neural network architecture employed is 25‐30‐40‐5. After training, the network was utilized for action signal classification, with the analysis of ANN efficacy conducted through accuracy assessment. In the case of Random Forest classification, data was categorized by generating multiple training sets and corresponding decision trees. Each decision tree evaluates the test data, culminating in comprehensive results that determine accuracy. The SVM utilizes a training set and a test set. Following normalization, the SVM classifier was trained with the training set and subsequently employed for prediction on the test set, yielding classification accuracy. The STFT involved the multiplication of time‐domain signals with a window function, followed by obtaining a local spectrum via the Fourier transform. The resulting time‐frequency graphs varied across the five groups of actions. By observing the signal variations and analyzing the characteristic parameters (such as signal strength, period, mean and variance, etc.), it provided features for machine learning algorithms.

### Characterization and Measurements

An electrical tensile tester (ZQ‐990B) and a digital multimeter were used for force and voltage measurements. The electrochemical properties of the samples were investigated using an electrochemical workstation (CHI‐CHI660E). The source meter (KEITHLEY‐2450) was used in constant current charge and discharge testing. The microstructure and morphology of the Mn/Ag interdigital electrodes and H_3_PO_4_/PVA hydrogel were characterized using SEM (TESCAN‐TESCAN MIRA). Elemental distributions at the Mn and Ag interdigital electrodes were analyzed via EDS. Furthermore, the functional groups in the samples were analyzed using Raman spectroscopy (HORIBA‐LabRAM HR Evolution) and FTIR spectroscopy (Thermo‐Nicolet iS20).

All procedures involved in wearable sensor testing were approved by the Ethics Committee of Qingdao University (20240118SD0320240120151). All volunteers provided informed written consent before participating in the study.

## Conflict of Interest

The authors declare no conflict of interest.

## Supporting information

Supporting Information

Supporting Information

Supporting Information

Supporting Information

Supporting Information

## Data Availability

The data that support the findings of this study are available from the corresponding author upon reasonable request.
