# Supplementary material for: Self‐Powered Biomimetic Pressure Sensor Based on Mn–Ag Electrochemical Reaction for Monitoring Rehabilitation Training of Athletes
Source: Adv Sci (Weinh). 2024 Apr 23;11(25):2401515. doi: 10.1002/advs.202401515 (PMC11220713; doi:10.1002/advs.202401515)
Supplement: Supplementary file 1 — Supporting Information [file ADVS-11-2401515-s005.pdf]

ADVANCED SCIENCE
Open Access

Supporting Information

for *Adv. Sci.*, DOI 10.1002/advs.202401515

Self-Powered Biomimetic Pressure Sensor Based on Mn–Ag Electrochemical Reaction for Monitoring Rehabilitation Training of Athletes

Ziyan Yang, Qingzhou Wang, Huixin Yu, Qing Xu, Yuanyue Li, Minghui Cao, Rajendra Dhakal, Yang Li* and Zhao Yao**

Supporting Information

Self-Powered Biomimetic Pressure Sensor Based on Mn-Ag Electrochemical Reaction for Monitoring Rehabilitation Training of Athletes

Ziyan Yang, Qingzhou Wang, Huixin Yu, Qing Xu, Yuanyue Li, Minghui Cao, Rajendra Dhakal, Yang Li*, and Zhao Yao**

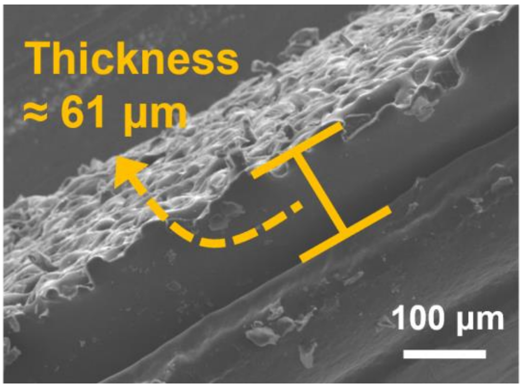

Figure S1. The SEM image of PVA/H_3PO_4 hydrogel electrolyte cross-section.

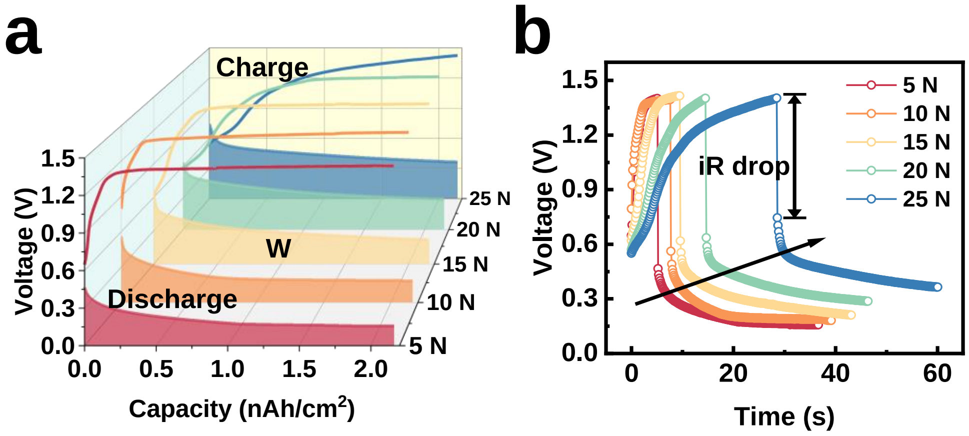

Figure S2. The constant current charge and discharge test.

The constant current charge-discharge test was conducted, and the results are illustrated in the Figure S2. The sensor exhibited stable charging and discharging capabilities throughout the test. Specifically, a charging cut-off voltage of 1.5 V, a discharge cut-off voltage of 0.1 V, and a constant current of 100 nA were employed. The charging process reveals a clear upward trend in the area-specific capacity[1] as the applied force gradually increases from 5 N to 25 N, as depicted in Figure S2a. Moreover, the output energy[2] (*W*) is quantified by the area under the integrated discharge curve, which can ·be determined as below:

$$W = \int_0^{t_d} IU(t)dt \tag{1}$$

where I is the cell current, U is the cell voltage, and t_d is the end time of discharging. During the discharging process, as the applied pressure increases, there is a corresponding augmentation in the output energy. Consequently, this sensor exhibits an enhanced capacity to store and release energy within a confined region.

The iR drop refers to the voltage decrease in a battery caused by the current passing through its internal resistance.[3] As depicted in Figure S2b, an increase in pressure leads to a reduction in both iR drop and internal resistance, which aligns with the previously established conclusion.

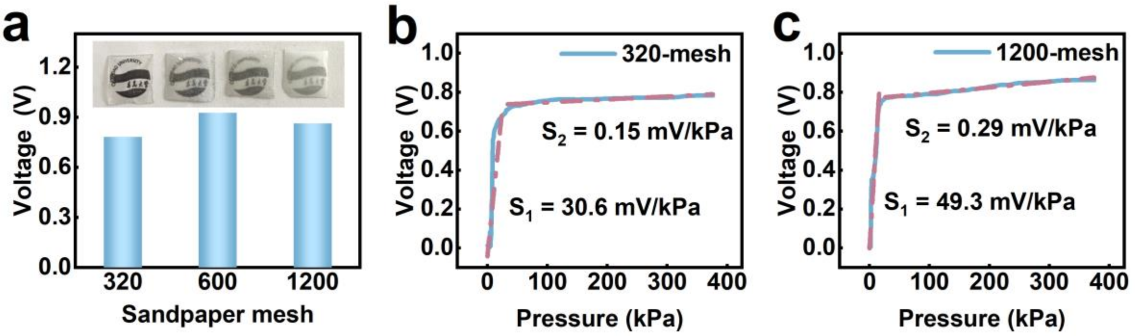

Figure S3. a) The maximum output voltage of hydrogel devices of different mesh sizes. b) Sensitivity plot of a 320-mesh hydrogel device. c) Sensitivity plot of a 1200-mesh hydrogel device.

The comparison of the transparency of hydrogels without structure and with different sandpaper mesh sizes is shown in Figure S3a. It can be seen that the transparency of the hydrogel decreases as the mesh size of the sandpaper increases. Figure S3b and c show the detail sensitivity plots of the sensor fabricated by 320-mesh and 1200-mesh, repectively.

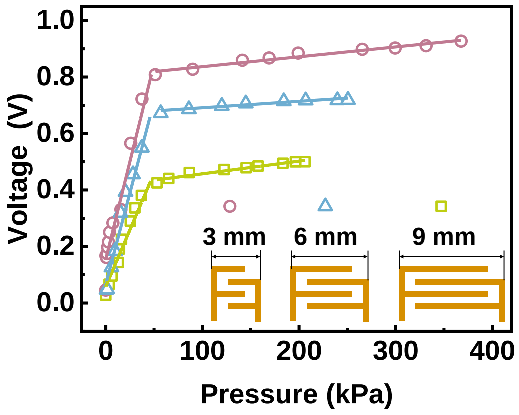

Figure S4. The sensitivity curves for different electrode lengths.

The impact of electrode size on sensor performance was investigated. The lengths of electrodes including 3, 6 and 9 mm were employed as experimental subjects. As the electrode length increased, there was a corresponding increase in the overall device area (80, 120 and 150 mm^2), while maintaining a maximum pressure of 30 N during testing. Consequently, it leads to a decrease in pressure levels when increase the electrode length. Therefore, the extended electrode length results in a decreased detection range of the sensor and a decreased maximum output voltage, as shown in Figure S4.

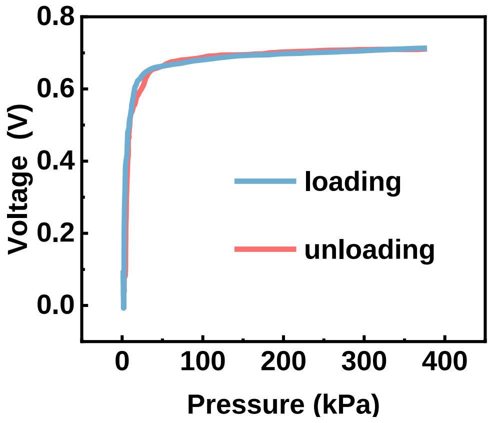

Figure S5. The hysteresis curve of the fabricated sensor.

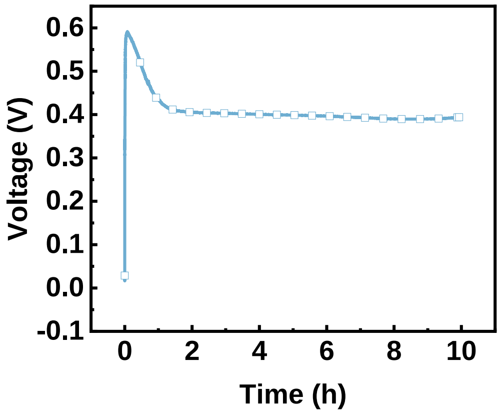

Figure S6. The prolonged pressing test of the fabricated sensor.

The sensor's performance was thoroughly investigated through continuous pressure for nearly 10 hours. The voltage exhibited a noticeable decline dropped from 0.59 V to 0.41 V within the initial·1.5 hours. However, throughout the subsequent testing period from 1.5 hours to 10 hours, the sensor's voltage exhibited surprising stability with only a decrease of about 0.02 V. The results of this study demonstrate the robust performance of the sensors under long-term sustained pressure.

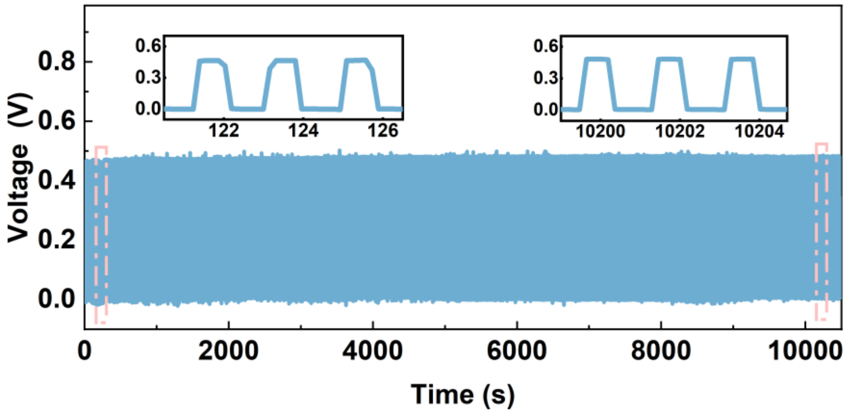

Figure S7. The cyclic response of the fabircated sensor.

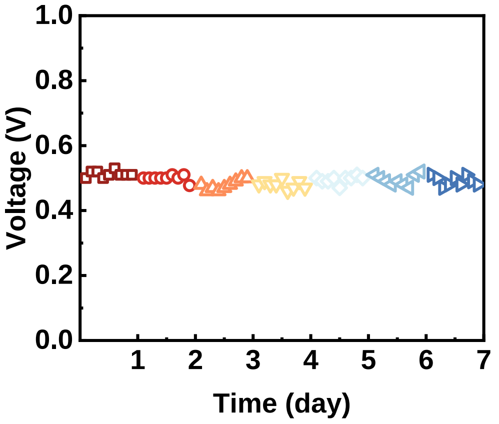

Figure S8. The long-term stability of the fabricated sensor.

The sensors were tested continuously for seven consecutive days, with hourly intervals, to assess the long-term stability. Within this period of seven days, the voltage drop observed in the sensor was less than 0.1 V, indicating a favorable level of long-term stability.

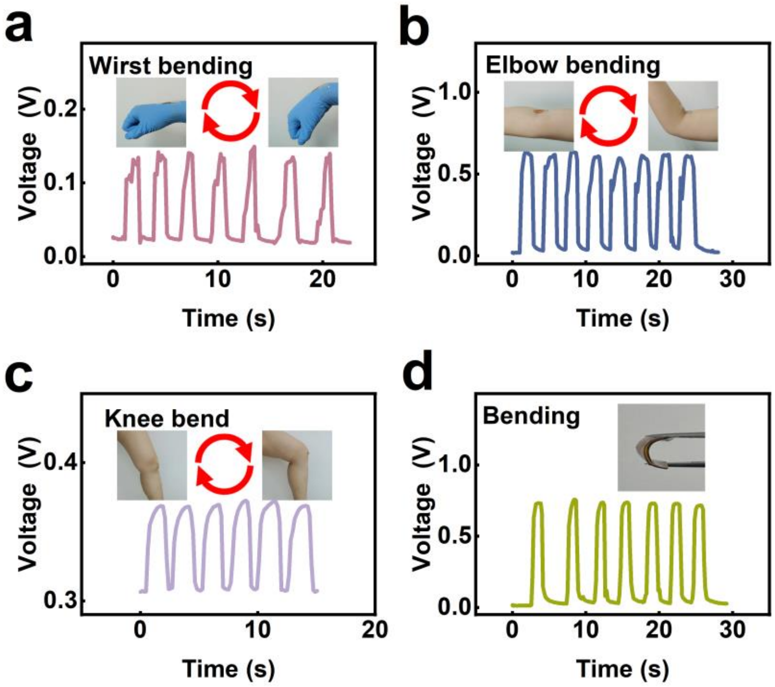

Figure S9. Sensor response curves for a) wrist flexion, b) elbow flexion and c) knee flexion, d) bending.

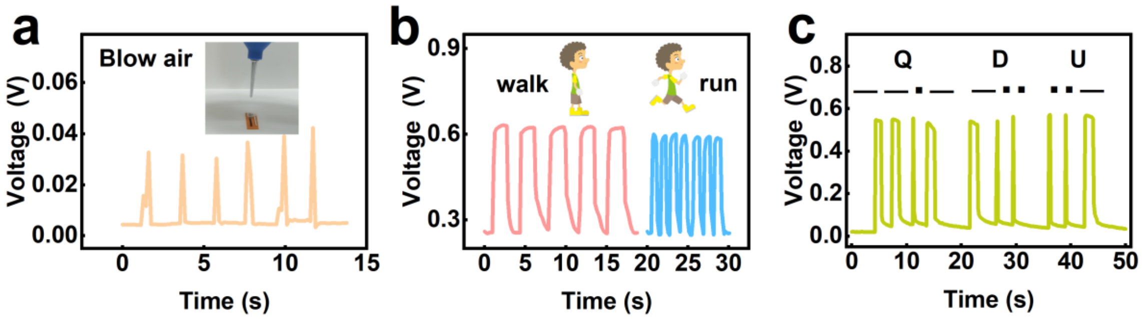

Figure S10. a) Sensor response curve for blowing. b) Sensor Response Curve for Walking and Running. c) QDU Morse code response curve.

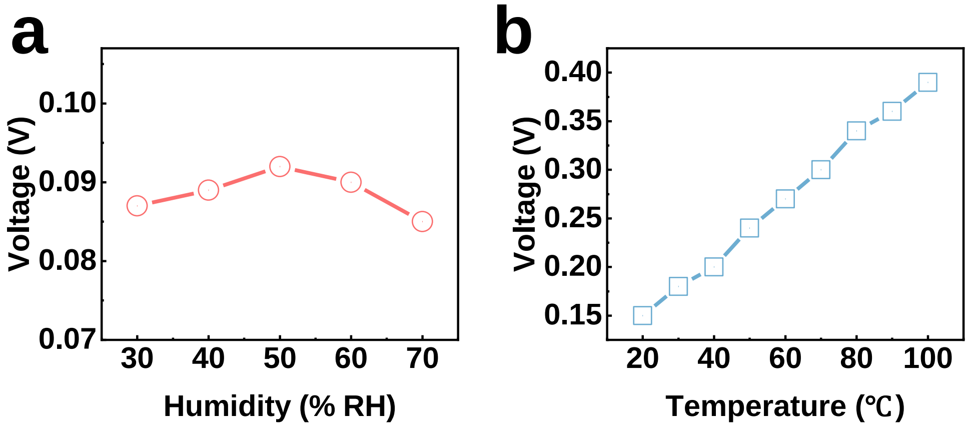

Figure S11. The response curve of the sensor to humidity and temperature.

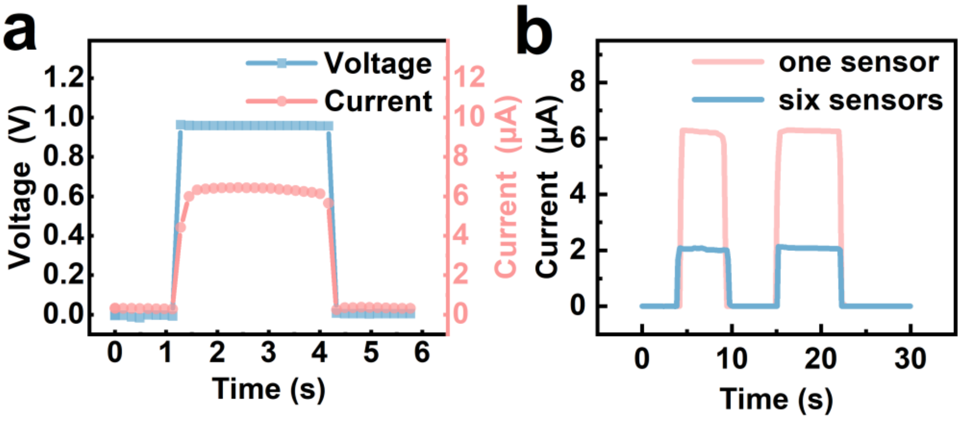

Figure S12. a) Current and voltage output response of a single sensor. b) The current output response of a single sensor and six sensors connected in series.

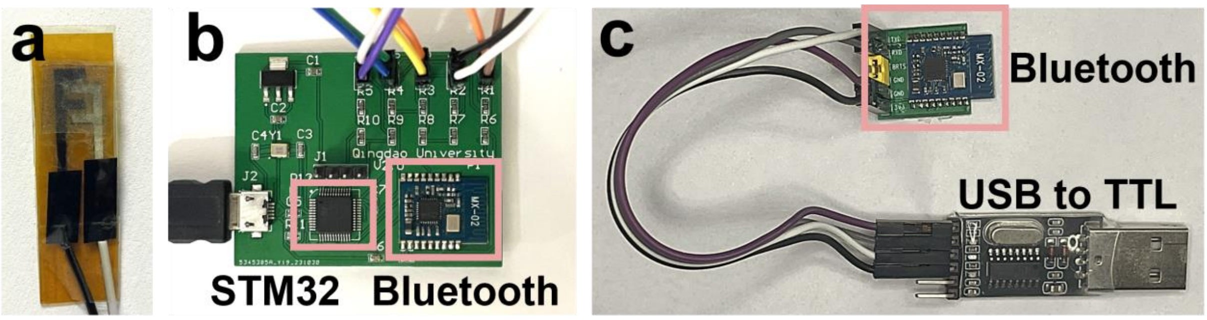

Figure S13. a) Optical image of the packaged sensor. b) Data acquisition system: PCB with STM32 and Bluetooth chips. c) Bluetooth and serial modules connected to the PC side.

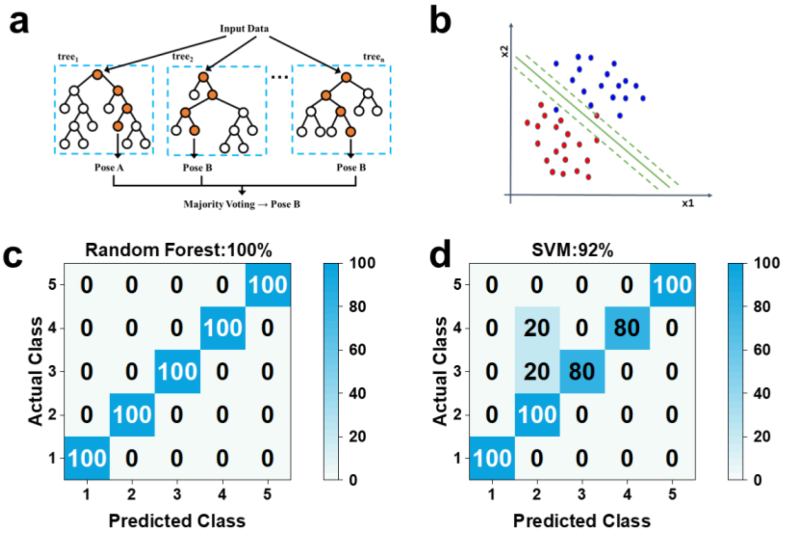

Figure S14. a) Schematic of the Random Forest algorithm. b) Schematic diagram of the SVM algorithm. c) Prediction result graph of Random Forest algorithm. d) Prediction result graph of the SVM algorithm.

Table S1. The sensing performance of the proposed sensor and the current state-of-the-art potentiometric pressure sensors.

Voltage	Current	Power density	Sensitivity	Reference
1.062 V	-	-	182.3 mV/N<br>(or 3.64 mV/kPa)<br>(0-1 N)	[24]
0.648 V	-	-	-	[27]
1.4 V	1000 μA	0.52 mW cm^{-2}	4.92 mV/kPa<br>(2-140 kPa）	[29]
0.58 V	3.2 μA cm^{-2}	-	-	[30]
0.77 V	2.4 mA cm^{-2}	1.93 mW cm^{-2}	-	[31]
0.28 V	-	-	205.5 mV/N<br>(0-1 N)	[33]
0.927 V	6 μA	0.34 mW m^{-2}	14 mV/kPa<br>(0-50 kPa)	This work

Supplementary Movie

SV1 is a video showcasing the functionality of the device group in emitting an SOS signal. According to the different lengths of pressing, the LEDs show the international distress signal SOS (three short, three long, three short).

SV2 is the video illustrating the process of charging a capacitor to illuminate a small lamp. The device set is connected to both ends of the capacitor and pressed to charge the capacitor. Subsequently, the charged capacitor is linked to an LED light through a single-pole double-throw switch. When the switch is closed, the capacitor supplies power to the LED light, thereby illuminating the small light.

SV3 is the video illustrating the process to energize the electronic counter using the proposed sensor. The device set is connected to the electronic counter, enabling the user to activate the electronic counter by applying pressure.

SV4 is the video showcasing the rehabilitation monitoring system. The five devices are attached to the limb joints and elastic band, when the human body makes different movements for rehabilitation, the signals are transmitted to the computer via Bluetooth in the back data acquisition system and displayed in LabVIEW.

[1] X. M. Yang; A. L. Rogach, Adv. Energy Mater. **2019**, 9 (25), 1900747.
[2] J. Landfors, *J. Power Sources* **1994**, 52 (1), 99-108.
[3] A. Hess; Q. Roode-Gutzmer; C. Heubner; M. Schneider; A. Michaelis; M. Bobeth; G. Cuniberti, *J. Power Sources* **2015**, 299, 156-161.
